# Foreign Medical Literature

**Published:** 1819-01-01

**Authors:** 


### THIRD DEPARTMENT.

### Foreign Medical Literature.

### ON ABSORPTION.

By F. MAJENDIE, Docteur in Medecine, &c.

(Precis Elementaire.)

Professor Majendie must be already known to all our readers as one of the most zealous experimenters and distinguished physiologists of the age. The two volumes which he has recently furnished upon the Elements of the Science form a most important contribution to our present knowledge. The last of these volumes in particular, is singularly valuable from its embracing and discussing nutrition, with the various functions subservient to it; and it is the laudable accuracy and philosophical cautiousness with which he has executed this part of his task, that will, we think, form his best title to fame.

While, then, we strongly recommend the whole of the volume to the study of our readers, we mean to limit our present review to his opinions about Absorption, and shall translate a few paragraphs in which some new and interesting conclusions are brought forward on that subject. This we are induced to do because the doctrine of Absorption is not merely a speculative question—not merely interesting as a matter of physiology; but is fundamentally connected with the *pathology* of dropsy and several other diseases, and has a reference equally intimate and important to the *cure* of them.

In order to clear the way to the question, and to prepare our readers for Dr. M's conclusions, we shall present a slight sketch of the history of the doctrine.—It was the opinion both of Hippocrates and Galen, that Absorption takes place as well from the skin as from the great cavities of the body. Indeed, the former expressly says, that inhalation, as well as exhalation, takes place from every part of the human frame.* This idea, along with other parts of Grecian knowledge, seems to have passed to the Arabian Physicians, and to have been adopted by them; for we find Avenzoar, one of the most distinguished of their medical writers, main-

---

* We shall subjoin the original words, to bear witness that we have not misrepresented their import, "Εάρκες όλκοι · και εκ κοιλιης και έξαθεν· δηλον ή αισθησις ώς εκπνοεν και εισπνοον όλον το σωμα."—HIPPOC. Op. Popul. Lib. 6. Sect. 6. Edit. Vand. Linden.

taining the opinion, and in conformity with it, recommending baths formed of milk or other nutrient fluids to be habitually employed in cases of Dysphagia.

Among these authors, however, as well as amongst all the other ancients, it was believed that absorption, whether from the skin or from the great cavities, takes place entirely by means of the veins and arteries; and this opinion obtained universal acquiescence until Azelius made the important discovery of the lacteals. About the middle of the last century, Dr. Munro, *secundus*, and Dr. William Hunter, without any concert or knowledge of each other's labours, discovered and described the lymphatics throughout nearly the whole body: and since that time physiologists, with few exceptions, have adopted the belief that absorption is exclusively performed by this newly-discovered system of vessels.

Recently, to be sure, the experiments and reasonings of Mr. Ellis*, which go to prove that air is not absorbed by the surface of plants, and which thence, by analogy, seem to disprove the absorption of gaseous substances by the skin (an opinion advanced by the distinguished Mr. Abernethy, in his Surgical and Philosophical Essays, as the reeult of *his* experiments)—and still more recently, the experiments of those very able physiologists, Sir Everard Home† and Mr. Brodie, proving that substances can find their way into the blood without the aid of either the lacteal or lymphatic absorbent vessels, have tended to throw great *doubts* at least, upon what, a few years ago, was considered an established doctrine in physiology.

Such was the state of the question when Professor Majendie took it up; and we now proceed to relate his experiments and conclusions.

" Though hitherto considered as true, it has been by no means proved that the fluids of the serous membranes and cellular tissue are absorbed by the lymphatic vessels, and transported by these vessels into the venous system. This opinion contains two distinct ideas; viz. 1st. That the lymph exists in different parts of the body, and 2d. That the lymphatic vessels are endowed with the faculty of absorbing it. Of these two notions, the former is quite inaccurate, and the latter demands particular investigation. In fact, although there is an analogy in point of appearance between the fluids observed on the surface of serous, cellular, or synovial membranes and the lymph, I shall prove that these fluids differ in their mechanical and chemical qualities. Now, as these serous, synovial, and cellular fluids differ so much from each other, if we admit them to be the source of the fluid found in the lymphatics, it follows we should observe several varieties of lymph. But so far as the latter fluid has hitherto been examined, it has always been found similar, in its sensible properties, in every part of the body." P. 170 and 177.

---

* An Inquiry into the changes induced on atmospheric air, &c. By Daniel Ellis. Chap. 2.

† Philosophical Transactions, 1811 and 1816.

The author then enters at great length into the experiments of Mr. John Hunter, instituted to prove absorption by the lymphatics, examines them critically, and labours to fix upon them a suspicion of inaccuracy. He asserts that he himself and others have repeated these very experiments with a result diametrically opposite to that detailed by our great physiologist; and that in those cases where the lymphatics surrounding abscesses have been found full of pus; instead of hastily concluding that it was absorbed from the abscess, it would be more probable to believe that it proceeded from inflammation and suppuration of their own coats; more especially as the pus has never yet been found to have passed the first lymphatic gland in its way.

Moreover, Dr. M. believes that the lymph is merely a part of the blood, which, instead of returning to the heart by the veins, follows the rout of the lymphatics; the extremities of these, he contends, inosculate with the extreme arteries, and that their calibre excludes the red, while it admits some of the serous part of the blood.

This may seem to our readers to annihilate altogether the function of absorption; but as its existence is a *fact*, explain it how we may; a fact, proved by the rapid and melancholy operation of animal poisons, and of many other deleterious substances, the author proceeded to make experiments upon the subject; and he soon saw reason to come to the conclusion that the *veins* are the real absorbents; he says,

"The venous twigs not only receive the blood of the ultimate arterial branches, but they present also another phenomenon sufficiently remarkable. All kinds of gas, or of liquid, placed in contact with the different parts of the body (except the skin) pass quickly into the small veins, and are soon carried forward to the lungs with the venous blood. The same thing happens with solid substances that are capable of dissolving in the blood or the fluids secreted from it. In a very short time these substances are absorbed by the veins, and carried to the heart and lungs." P. 229.

"In order to have an idea of this property, which is common to all the veins, we have only to introduce a solution of camphor into one of the serous or mucous cavities of the body; or we may thrust a morsel of solid camphor into the tissue of any organ. In a few minutes the air expired from the lungs will manifest a very palpable odour of camphor. The same may be observed when we employ any other odorous body which does not combine with the blood."

"The corrosive quality of liquids or solids used in the above manner does not hinder them from being absorbed, (though physiologists have hitherto taught that the mouths of the absorbents have a peculiar elective sensibility, which enables them to resist noxious fluids applied to them,) on the contrary the absorption of such substances is more ready than of those innocuous solids or fluids which do not act upon the tissues." P. 230.

The learned author then goes on to state that the *villi* in the different parts of the intestinal canal are formed in part by venous twigs, which absorb all the fluids in the intestines, with the single exception of the chyle (this last is absorbed by the lacteals only, and finds its way into the blood through the thoracic duct), and that these fluids are carried to the heart and lungs directly through the venæ portæ, whose function he conjectures to be to minutely subdivide and mix with the blood the fluids thus absorbed, lest they might prove detrimental, as they inevitably would, if carried to the heart without this minute division and intermixture.

He contends that the skin, *while its cuticle remains entire*, does not absorb any substance in contact with it; but if this is abraded, or if the substance employed acts chemically and corrodes the epidermis, or if the particles of the substances used are, by friction, made to penetrate under the squamæ of the cuticle, into those minute openings through which the insensible perspiration proceeds, that then absorption takes place by the minute veins the same as in any other texture of the body. These conclusions he elucidates by pointed experiments, which, so far as we can judge, seem accurate and unobjectionable.

We shall here transcribe one of his chief experiments, not merely as to the fact of absorption by the skin when abraded of its cuticle, but as to the general fact of absorption taking place *exclusively* by the veins:

" M. Delille and I separated the thigh from the body in a dog which had been previously rendered insensible by opium, in order to spare it the pain attending so laborious an operation. We left the limb attached by nothing but the crural artery and vein. These two vessels were dissected with the utmost care; that is, they were isolated to the extent of nearly three inches: their cellular coat was removed, lest it might conceal some lymphatic vessels. Two grains of a very subtle poison *(upas tiente)* were then forcibly thrust into its paw. The effects of this poison were quite as immediate and intense as if the thigh had not been separated from the body; so much so that they displayed themselves before the fourth minute, and the animal was dead before the tenth.

" It might have been objected, that in spite of all the precautions taken, the coats of the crural artery and vein still contained lymphatics, and that these vessels were enough to afford a passage to the poison.

" To remove this difficulty, I repeated upon another dog the preceding experiment, with this modification: I introduced a small barrel of a quill into the crural artery, and fixed the vessel upon it by two ligatures. The artery was immediately cut all round betwixt the two ligatures.

" I adopted precisely the same proceeding with the crural vein. Consequently there was no longer any communication between the thigh and the rest of the body, except by the arterial blood which came to the thigh, and the venous which returned to the trunk.

Yet the poison introduced into the paw, produced its effects in the ordinary time, that is to say, at the end of about four minutes.

"This experiment leaves no room to doubt that the poison had passed from the paw to the trunk by the crural vein. To render this phenomenon still more evident, it is necessary to compress this vein between the fingers at the moment when the effects of the poison begin to be developed: these effects speedily cease. They reappear when we leave the vein free, and cease if the vein is compressed afresh. In this way we can regulate the effects at our pleasure." P. 238.

Professor M. supports his favourite doctrine of venous absorption by argument as well as by experiment. He reminds us that the most minute anatomy has not enabled any one to discover lymphatics in the brain, the eye, or the placenta, although we have convincing proof that absorption takes place from these organs with as much promptitude as in other parts. Moreover, animals without vertebræ, which have blood, possess no lymphatics; yet in them absorption is manifest. And lastly, the thoracic duct, he thinks, is much too small to give a ready passage to the matters absorbed in every part of the body, but particularly to our drink. All these facts, he contends, are perfectly capable of explanation, when once absorption by the veins is admitted.

His opinions may be summarily comprehended under three propositions; viz. 1. That it is certain the lacteals absorb the chyle. 2. That it is improbable they absorb any thing else: and, 3. That it is not demonstrated that the lymphatics are endowed with an absorbent power, and that it is proved that the veins enjoy this property. Upon the whole, these conclusions are very important; and as the experiments seem unobjectionable in every respect, we should have little hesitation in giving them our entire confidence, did we not recollect, that even experiment itself is fallacious: for what else can we say, when we perceive the very same experiments in the hands of physiologists, equal in acuteness and in industry, producing results directly contrary?

The subject of cutaneous aborption has been of late years a subject very prolific of discussion, nor does that discussion seem likely to be soon terminated. We have at this moment before us an interesting treatise on the subject by Dr. Young.* This gentleman states pretty copiously the arguments for and against the doctrine, and relates twelve experiments, which seem to have been accurately conducted, and of which he himself was the subject. On using a bath for an hour, the water being of the temperature of 80°, he found the weight of his body increased by five ounces, and the secretion of urine augmented: if the bath was used at 100°, or above it, he found that his body neither lost nor gained in weight, but that the copious perspiration from the stimulus of heat counterbalanced the water absorbed. He also anointed his body with ol. terebinth. and other substances, and detected the violet smell in the

* Dissert. Inaug. de Cutis Inhalatione Auct. N. L. Young. Edinb. 1817.

urine; though in this, as in all his experiments, he breathed the open air through an apparatus which prevented the possibility of the vapour of the warm baths, or the oil of turpentine, being absorbed by his lungs. In conclusion, he affirms the absorption by the skin, and applies it to explain the chief phenomenon of diabetes. As long as such striking differences in result exist, as between his and Dr. Majendie's experiments, the subject must be considered open to further investigation.*

* Many considerations have induced us to abridge our Department of Foreign Medical Literature, of which, however, we shall only state two in this place. First, our paramount reason is this; that we mean to present, through the medium of the Medico-Chirurgical Journal, a more comprehensive Analytical Review of *British* Medical Literature than has ever yet been presented in any periodical work. We appeal to the present Number as a specimen, and pledge ourselves that *all* succeeding Numbers of the Journal shall contain an equal, or a *greater* quantity of Analytical Matter. The effect of such a circulation of concentrated information through the minute ramifications of the profession, where expensive works cannot go, may be easily anticipated.

Secondly, To those who wish for more extended views of foreign medicine, we would recommend "THE QUARTERLY JOURNAL OF FOREIGN MEDICINE AND SURGERY," conducted by a Gentleman of talents in this metropolis, assisted by many able correspondents abroad.

